# A128 ANTIBIOTIC RESISTANCE DOES NOT FULLY EXPLAIN *HELICOBACTER PYLORI* TREATMENT FAILURES

**DOI:** 10.1093/jcag/gwae059.128

**Published:** 2025-02-10

**Authors:** T Krahn, L Turnbull, R Rennie, S Veldhuyzen van Zanten

**Affiliations:** University of Alberta, Edmonton, AB, Canada; Alberta Precision Laboratories, Calgary, AB, Canada; University of Alberta, Edmonton, AB, Canada; University of Alberta, Edmonton, AB, Canada

## Abstract

**Background:**

Most treatment failures of *Helicobacter pylori* are attributed to antibiotic resistance or patient nonadherence. Commonly used 1st-line treatment regimens include 14 day concomitant [proton pump inhibitor(P), amoxicillin(A), metronidazole(M), and clarithromycin(C):PAMC], or bismuth-based quadruple therapy [P, Bismuth(B), M, and tetracycline(T):PBMT]. Levofloxacin(L)-based PAL is also suggested for 14 days and rifabutin(R)-based PAR for 10 days. Analyses of data on the frequency of treatment failures in antibiotic sensitive cases are scarce.

**Aims:**

To determine the proportion of *H. pylori* treatment failures not explained by antibiotic resistance.

**Methods:**

Cultures of *H. pylori* positive patients (by histology, urea breath test, or stool antigen test) at the University of Alberta Hospital in Edmonton, Canada were assessed for resistance by E-test according to EUCAST thresholds to C, M, A, T, and L measuring minimum inhibitory concentrations.

**Results:**

There were 68 positive cultures from 64 individuals in 292 cases. Treatment adherence was high in patients with follow-up (FU) testing available. Median number of antibiotic regimens received prior to culture was 2 (IQR 0-3). In patients not previously treated, overall cure rate was 10/17 (59%) vs 21/47 (45%) for those who had been previously treated (p=.32). Summary data on outcomes are shown in Fig 1.

Clarithromycin: 14/63 (22%) cases had C-sensitive cultures. Regimens containing C were successful in 5/8 (63%), failures were PAC(1), Sequential therapy (1), and PMC(1). Of C-sensitive cases, 5/14 (36%) had been previously treated with C: further treatment success was 1/2 with PAR and 0/1 with PAL.

Metronidazole: 25/52 (48.0%) cases were M-sensitive: 11 cases were treated with M-containing regimens. 80% (8/10) were successfully treated with PBMT or PAMC, 1 case failed. Success with non-M regimens was 67% (4/6) with PAR and 1/2 with PAL. PBMT was successful in 60% (3/5) of M-sensitive patients with prior M-exposure.

43% (22/51) of cases had dual resistance to C and M. In 5 cases who were sensitive to both C and M, 60% (3/5) were cured with PAMC, 1 declined treatment and 1 was lost to FU.

Levofloxacin: 22/57 (39%) cases were L-sensitive of which 41% (9/22) had been previously treated with PAL. Successful treatment with PAL was 67% (4/6) in L-sensitive cases.

There were no cases of T-resistance (0/66). Borderline A-resistance was observed in 9% (4/46) of cases.

**Conclusions:**

*H. pylori* treatment failures are not fully explained by antibiotic resistance. A substantial proportion (27-41%) of patients failed despite being sensitive to the antibiotics used (C, M, L). M resistance can be partially overcome with combination treatment. Resistance to A and T is rare. Clinical history including adherence is an important complement to antibiotic resistance testing in *H. pylori*, especially to determine previous C exposure.

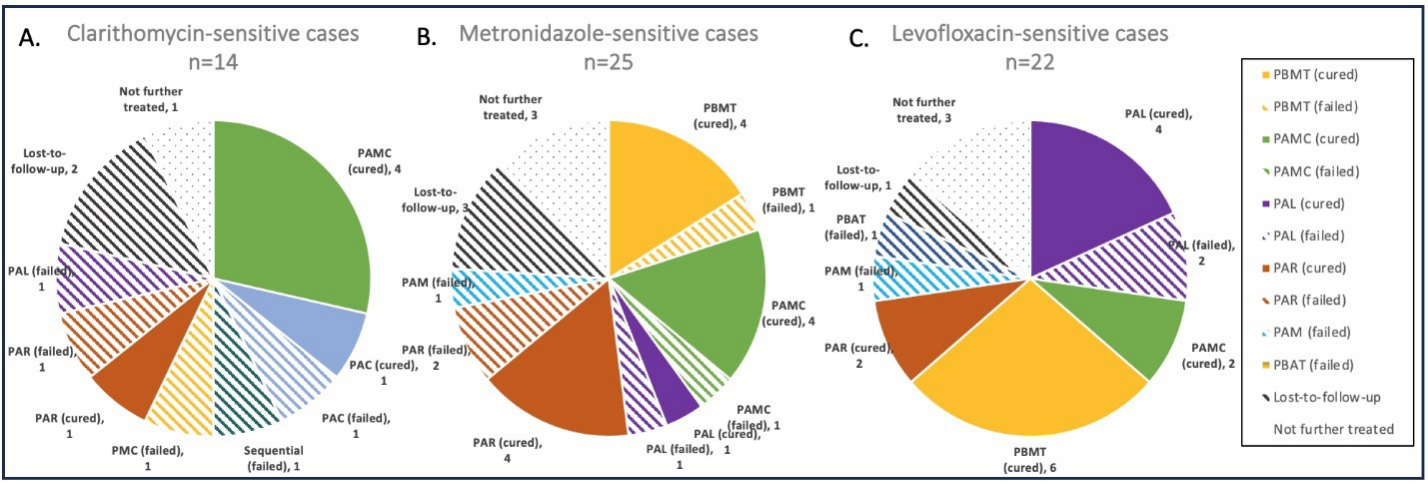

Figure 1: Treatment success in confirmed cases with antibiotic-sensitive *H. pylori* cultures.

Abbreviations: PPI, proton pump inhibitor; PAMC, PPI-amoxicillin-metronidazole-clarithromycin; PAC, PPI-amoxicillin-clarithromycin; PMC, PPI-metronidazole-clarithromycin; PAR, PPI-amoxicillin-rifabutin; PAL, PPI-amoxicillin-levofloxacin; PBMT, PPI-bismuth-metronidazole-tetracycline; PAM, PPI-amoxicillin-metronidazole; PBAT, PPI-bismuth-amoxicillin-tetracycline

**Funding Agencies:**

None

